# Gender differentiated small-scale farm mechanization in Nepal hills: An application of exogenous switching treatment regression

**DOI:** 10.1016/j.techsoc.2020.101250

**Published:** 2020-05

**Authors:** Gokul P. Paudel, Hom Gartaula, Dil Bahadur Rahut, Peter Craufurd

**Affiliations:** a International Maize and Wheat Improvement Center, South Asia Regional Office, P O Box: 5186, Kathmandu, Nepal; b International Maize and Wheat Improvement Center, G2, B Block, National Agricultural Science Centre Complex (NASC), Dev Prakash Shastri Marg, New Delhi, 110012, India; c Socioeconomics Program, International Maize and Wheat Improvement Center, El Batan, Mexico; d Sustainable Intensification Program, International Maize and Wheat Improvement Center, South Asia Regional Office, P O Box: 5186, Kathmandu, Nepal

**Keywords:** Female-headed households, Mini-tiller adoption, Exogenous switching treatment regression, Market access, Nepal hills

## Abstract

Farm mechanization among smallholder farming systems in developing countries is emerging as a viable option to off-set the effects of labor out-migration and shortages that undermine agricultural productivity. However, there is limited empirical literature on gender and farm mechanization. This study assesses the impacts of the gender of household heads on mini-tiller adoption in the hills of Nepal, using an exogenous switching treatment regression model. Our findings reveal that there is a significant gender gap in mini-tiller adoption between male-headed households (MH-HHs) and female-headed households (FH-HHs). Compared to MH-HHs, the mini-tiller adoption rate is significantly lower among the FH-HHs, and a large amount of unobserved heterogeneity is deriving this difference. Moreover, when MH-HHs and FH-HHs have similar observed attributes, the mini-tiller adoption rate among the food insecure FH-HHs is higher than in the food secure group. The gender-differentiated mini-tiller adoption rate can be minimized primarily by enhancing market access. Findings suggest that farm mechanization policies and programs targeted to the FH-HHs can reduce the gender-differentiated adoption gap in Nepal and similar hill production agro-ecologies in South Asia, which will enhance the farm yield and profitability.

## Highlights

• Farm mechanization is significantly different between MH-HHs and FH-HHs.
• Mini-tiller adoption rate is significantly lower among the FH-HHs.
• Substantial amount of unobserved heterogeneity is affecting the adoption difference.
• Food insecure FH-HHs would benefit most from mechanization intervention.
• Mini-tiller adoption difference between MH-HHs and FH-HHs can be minimized by enhancing market access.

## 1 Introduction

Farm mechanization is often heralded as a key agricultural approach to achieve the economy of scale, off-set the effects of labor shortages, reduce crop cultivation related drudgeries, and enhance yield and profitability [[1], [2], [3]]. In developing countries, poverty and smaller farm size make the challenge of farm mechanization unique [[4], [5], [6]]. In recent years, the use of small-scale mechanization among smallholder farming systems in South Asian and African countries is increasing, mainly through service provision, indicating positive signs of agricultural transformation [5,[7], [8], [9], [10], [11], [12], [13]]. Nevertheless, most of the existing farm mechanization-based technologies are, unfortunately, either gender blind or at most gender-neutral - to the detriment of women - and minimizing gender disparity among smallholder farmers has been a primary concern for planners and policymakers. In this situation, achieving the goal of gender equality (SDG5) by 2030, as highlighted in the sustainable development goals (SDG) of the United Nations, is a big challenge [14].

The history of farm mechanization started with the advent of the green revolution in the mid-twentieth century. The benefits gradually trickled down to developing countries with the colonial legacy of technology transfer in the 1960s and 70s and with the introduction of new seed varieties requiring heavy equipment such as tractors, trucks, water pumps, reapers, threshers, and combine harvesters [15]. Farm mechanization, historically, has focused on high input large-scale agriculture promoted by green revolution rather than on smallholder subsistence agriculture. However, scale-appropriate farm mechanization is receiving more attention now [4,5,10,11], with greater importance on sustainability and equity [9,16,17].

In developing countries, most women are marginalized, and they have limited access to and control over resources like land, information, markets, education, extension services, and agricultural credit [[18], [19], [20], [21]], which adversely affects the adoption of agricultural technologies, including farm mechanization. Therefore, closing gender inequalities is likely to enhance the adoption of mechanization and other agricultural technologies in developing countries. Nonetheless, even after closing the gender gap and having the same level of access to and control over the household assets, adoption of farm mechanization may differ between male-headed households (MH-HHs) and female-headed households (FH-HHs), due to differences in the societal perception to consider women as farmers [22]. For example, FH-HHs may have similar years of education level, land entitlements, credit access, and so on, but established societal norms may prevent women from tilling the land using machines. Under such a situation, the adoption rates of farm mechanization for the MH-HHs and FH-HHs would be diverse until these deep-rooted and socially established beliefs are changed through policy interventions. Another example that could bring differential farm machinery adoption across MH-HHs and FH-HHs is the operationalization difficulties associated with farm machinery. Farm machinery often requires high physical effort and women may have lower physical strength [23,24] to operate such heavy machines.

Importantly, MH-HHs and FH-HHs are also not a homogenous category; they differ among themselves in many dimensions due to socially established norms and values that may differ across societies [25]. However, using gender as a single dummy variable in the pooled regression is a common approach for assessing the impacts of gender inequality (see, for example [26]), which is associated with several problems to yield meaningful results. First, gender, as a single dummy variable in the pooled regression, does not account for the interactions between gender and other farm-level attributes. Second, the difference among MH-HHs and FH-HHs cannot be captured by including gender as a single dummy variable because of the effects of potential unmeasured heterogeneity such as the difference in the quality of resources, experience with farm machinery, or differential access to machinery services, and families societal background, etc. The results on the estimated impact solely rely on the observed attributes and undermine the unobserved sources of heterogeneity. Hence, it is important to use separate regressions for MH-HHs and FH-HHs. Furthermore, measuring some of the gender-related disparities are impossible, even using farm or household surveys as data collection tools, as surveys can only record the observed attributes among MH-HHs and FH-HHs that may potentially affect the farm machinery adoption decisions.

In this context, this study provides unique evidence on gender-differentiated small-scale farm mechanization, thereby accounting mini-tillers adoption in the hills of Nepal.^1^ It uses the exogenous switching treatment effect regression to compare the scenarios wherein household headship changes between male to female and vice versa, with all the relative attributes for adopting mini-tiller (see more in the methods section). This study overcomes the limitation of not having intra-household gender-differentiated data. We also assess the differences in mini-tiller adoption rates as affected by such unobserved heterogeneities that drive differential adoption rates. The study contributes to the current gender research in three different aspects. First, as per our literature review, this is the first study to investigate the gender-differentiated adoption of farm mechanization among smallholders in a developing country. Second, unlike the traditional empirical methods, we use an exogenous switching treatment effects, which appraise the heterogeneity effects of male- and female-headship on mini-tiller adoption, which is a better estimate for gender comparison. Third, we decomposed the gender-differentiated farm mechanization adoption across households’ food security to analyze the adoption effect of mechanization across families by food security cohorts.

## 2 Background of farm mechanization in Nepal

Nepal is a predominantly agrarian country, with almost 65% of people relying on farming, contributing to over a quarter of the countrywide economy [27,28]. However, due to the recent phenomenon of men leaving agricultural jobs and taking non-agricultural jobs (mostly outside the country), the agricultural sector is increasingly feminized [[29], [30], [31], [32]]. This has resulted in a state where women are required to take up additional tasks of farming in the inherently male-dominated sector [30,32,33]. In this changing situation, gender-responsive farm mechanization would not only save their time and efforts but also empower them through skills enhancement and farm management.

The shortage of agricultural laborers, primarily due to male labor out-migration, has been the driver of farm mechanization in Nepal [5,31,34]. However, most technologies, including farm mechanization, are traditionally considered the domain of men, and women are less involved in technology selection and adoption processes [35]. On another front, the scarcity of labor has raised wages, especially for male laborers [[36], [37], [38], [39]]. Due to out-migration and rise in the wage of male laborers, farming has become more challenging, especially for FH-HHs who have limited access to and command on the household and community productive assets [18]. Hence, some cultivable land has been converted to fallows [40,41]. Farm mechanization-based interventions can attenuate such problems of labor shortage, if women would be able to adopt agricultural technologies. This will not only help in timely cultivation of crops and improve productivity, but also enhance the well-being of FH-HHs by providing them access to machinery and reducing the cost and drudgery of cultivation.

The challenging landscapes of Nepal – particularly the hilly areas – therefore requires the improvement and scaling of technologies which not only saves time, effort and cost, but also supports women's empowerment and reduce gender inequality. Recognizing the importance of the role of female members in farming and development would contribute to achieving the sustainable development goals (SDG) of the United Nations by 2030 [14]. Policymakers in Nepal have recognized small-scale mechanization as an important intervention to substitute labor scarcity. The Government of Nepal propagated an agricultural farm mechanization policy in 2014, subsequently following the formulations of several other related promotion policies [42,43]. These policies incorporated provisions of subsidies for promoting farm mechanization in Nepal [43]. However, these subsidy programs are largely beneficial to the MH-HHs due to FH-HHs low level of interactions with the extension agents and institutions. In this context, this paper compares the adoption of mini-tillers amongst MH-HHs and FH-HHs in the hilly regions of Nepal. Moreover, timely investigation of gender-differentiated adoption of agricultural machinery in the Nepal hills could contribute to the formulation and promotion of the gender segmented farm mechanization policies and programs, which would in turn have a significant role in improving the adoption level among female-headed households and maintain gender equality.

## 3 Materials and methods

### 3.1 Data

**Fig. 1:**
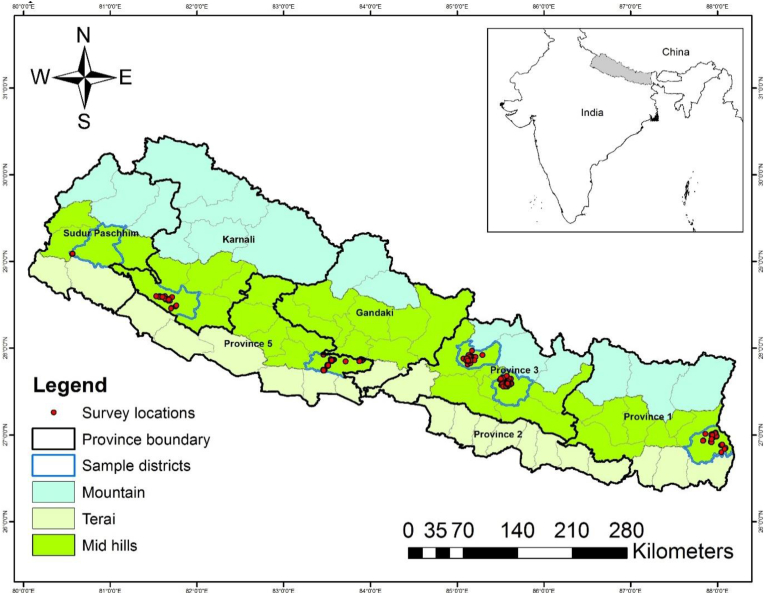
Map shows the sampled districts, sample locations, and Nepal hills.

This paper uses primary household survey data collected from the mid-hills of Nepal. In the mid-hills of Nepal, mixed crop-livestock farming system prevails, and maize is the primary crop grown for food, feed, and fodder in a rainfed upland area, while farmers also grow rice in the lowlands areas in the rainy season. However, in the winter season farmers also grow wheat, mustard, lentil, and vegetables. During the third quarter of 2017, a structured questionnaire was administered among 1004 men and women heads of households in the selected six districts of the Nepal mid-hills (Fig. 1). The districts were purposively sampled based on the higher level of mechanization, the distribution of mini-tillers adopted in the Nepal hills. From the six districts, 39 sub-districts were purposively selected based on the high spread of mini-tillers in each sub-district (also known as; Village Development Committees or VDCs).^2^ Finally, the sample households consisting of 841 (84%) MH-HHs and 163 (16%) FH-HHs were selected randomly for the survey. Out of the total samples of 163 FH-HHs, only 12 FH-HHs were the mini-tiller owner users and 18 were the renter (who rent the mini-tiller services from other owners) users. However, in the MH-HHs category, out of the 841 samples, 241 MH-HHs were owner users and 105 MH-HHs were renter users. In this study, we considered mini-tiller owner users and renter users as adopters and all the owners used their mini-tillers in their farms for agricultural land preparation. The survey used an electronic device called Surveybe (http://surveybe.com, retrieved July 30, 2019) to reduce post-collection data entry inaccuracies and the survey duration. Data were collected on the household demographics, socioeconomic profile, crops grown, incomes, household resources and expenditure, and agricultural inputs and outputs.

### 3.2 Empirical framework

In order to measure the impacts of the gender of household heads on farm mechanization, we could use a pooled regression with a dichotomous gender variable, i.e. MH-HHs or FH-HHs, which is the conventional approach. However, this will not account for the interactions between gender and household level attributes in the model. As the inclusion of gender dummy in pooled regression only estimates the intercept effect (i.e., homogenous shift in slope), which would not change despite the difference in the value of other household-level attributes affecting the adoption. Therefore, it fails to provide real scenarios related to gender-differentiated farm mechanization. To address this situation, we used an exogenous switching treatment effect regression (ESTER) in a counterfactual scenario to evaluate the cause and effects of mini-tiller adoption. Mini-tiller adoption is a dichotomous choice outcome indicator that indicates the adoption status, where the households are classified as mini-tiller adopting and non-adopting households. We tested the homogenous shift in the slope effects by applying Chow test, and the result indicated that it was essential to estimate household heads gender-specific coefficient since the statistical test of the null hypothesis was not accepted at 1% level of significance [χ^2^(19) = 38.37***, *p* = 0.003].

While using the ESTER, we estimated two separate equations for the MH-HHs and FH-HHs in our dataset.

(1) $$\left\{ \begin{array}{c} y_{m}=x_{m}\beta_{m}+u_{m}\;if\;g=1\\ \;\\ \;\\ y_{f}\;\;=x_{f}\beta_{f}+u_{f}\;\;if\;g=0\;\;\; \end{array}\right.$$

In Eq. (1), m and f represents the MH-HHs and FH-HHs respectively, g is the dichotomous choice variable, which is 1 if the head of the family is a man and 0 if the head is a woman. The variable y represents the outcome indicator. In our case, y represents the adoption status of mini-tiller, which is a dummy variable and represents 1 for adopter and 0 for non-adopter household. This dummy nature of the adoption variable provides the aggregated adoption rates across MH-HHs and FH-HHs from the entire sample. It also provides gender differential adoption rates that can be estimated using ESTER framework, i.e., what would have been the adoption rate of mini-tiller for FH-HHs if all the returns (coefficients) on their households' attributes had been the same as the coefficients of MH-HHs’ and vice versa? Any difference in the adoption rates while switching the coefficients of MH-HHs’ attributes to FH-HHs, and MH-HHs’ to FH-HHs, can be assigned to the gender differential adoption rate. The parameters x and β represent the vectors of household-level attributes and coefficients, respectively. The u represents a randomly distributed error term with mean zero and constant variance.

Based on the above described ESTER framework, the adoption status of mini-tiller is assessed by estimating the counterfactual scenario. The mini-tiller adoption status for each household is assessed by assigning the explanatory variables coefficient of MH-HHs to FH-HHs, and FH-HHs’ attributes coefficient to MH-HHs. This provides a procedure to get insights for the comparison between the expected adoption rates for mini-tiller adoption under actual and counterfactual scenarios as well as being useful to assess the impacts of gender on technology adoption. Moreover, following [44], the equations can be presented in the following ways:

(2) $$E\left(y_{m}|g=1\right)=x_{m}\beta_{m}$$

(3) $$E\left(y_{f}|g=0\right)=x_{f}\beta_{f}$$

(4) $$E\left(y_{f}|g=1\right)=x_{m}\beta_{f}$$

(5) $$E\left(y_{m}|g=0\right)=x_{f}\beta_{m}$$

where *E* is the anticipation operation. Eqs. (2), (3) are observed mini-tiller adoption status for the MH-HHs and FH-HHs, respectively. Eqs. (4), (5) are the counterfactual adoption status for the MH-HHs and FH-HHs, respectively. The use of these conditional anticipations combining gender as a treatment variable allows us to estimate the impacts of gender on mini-tiller adoption (Table 1).

**Table 1:** Conditional expectations, returns, and heterogeneity effects.

Types of households	MH-HHs	FH-HHs	Treatment effects
MH-HHs	$E\left(y_{m}|g=1\right)$ (a)	$E\left(y_{f}|g=1\right)$ (c)	MHHMT=(a – c)
FH-HHs	$E\left(y_{m}|g=0\right)$ (d)	$E\left(y_{f}|g=0\right)$ (b)	FHHMT=(d – b)
Heterogeneity effects	$BH_{m}$= (a – d)	$BH_{f}$=(c – b)	

Notes: (a) and (b) denote the mini-tiller adoption status for the MH-HHs and FH-HHs, respectively, while (b) and (c) denote the counterfactual mini-tiller adoption status for the MH-HHs and FH-HHs. The symbol g = 1 indicates the mini-tiller adoption in the MH-HHs, while g = 0 indicates the mini-tiller adoption for the FH-HHs. $BH_{m}$ and $BH_{f}$ indicate the difference in mini-tiller adoption rates for the MH-HHs and FH-HHs, respectively.MHHMT and FHHMT represent the expected mini-tiller adoption effects of gender for the MH-HHs and FH-HHs, respectively.

If MH-HHs’ attributes coefficient has comparable coefficients as those of FH-HHs, then the effect of gender on mini-tiller adoption is the difference between Eqs. (2), (4), which can be presented as:

(6) $$MHHMT=E\left(y_{m}\left|g=1\right)-E(y_{f}|g=1\right)=x_{m}\left(\beta_{m}-\beta_{f}\right)$$

Similarly, if FH-HHs’ attributes coefficient has similar coefficients as those of MH-HH, then the effect of gender on mini-tiller adoption is the difference between Eqs. (3), (5) that can be presented as:

(7) $$FHHMT=E\left(y_{m}\left|g=0\right)-E(y_{f}|g=0\right)=x_{f}\left(\beta_{m}-\beta_{f}\right)$$

The MHHMT and FHHMT give the expected mini-tiller adoption status for MH-HHs and FH-HHs, respectively. Eqs. (6), (7) are similar to the average treatment effects for the treated and untreated, respectively in the gender-related impact assessment research [[45], [46], [47], [48]].

However, MH-HHs and FH-HHs may have a different level of mini-tiller adoption status, even if they have similar observed attributes. MH-HHs may inherently be using mechanization more than FH-HHs (for example due to inherent skills, knowledge, risk-bearing capacity, etc.) and this may affect the adoption status, even if they have similar observed attributes and these can be referred as endogenous determinants of mini-tiller adoption or the base heterogeneity effects. The presence of base heterogeneity effect BH can be detected if there exists a significant deviation in the variance between Eqs. (2), (3), (4), (5) as:

(8) $$BH_{m}=E\left(y_{m}\left|g=1\right)-E(y_{m}|g=0\right)$$

(9) $$BH_{f}=E\left(y_{f}\left|g=1\right)-E(y_{f}|g=0\right)$$

## 4 Results and findings

### 4.1 Description of the variables

The description of variables used in the model, with their mean values and standard deviations across gender-disaggregated household headship, is reported in Table 2. The survey data show that the overall mini-tiller adoption rate in the hilly regions of Nepal is 37%.^3^ While the adoption rate for MH-HHs is 41% and only 18.5% for FH-HHs. This indicates that FH-HHs have substantially lower mini-tiller adoption rates, which could be explained by many household attributes that play a role in the adoption decision.

**Table 2:** Household-level attributes differentiated by MH-HHs and FH-HHs in Nepal hills.

	Overall households (N = 1004)		MH-HHs (N = 841)		FH-HHs (N = 163)		Difference (%)
	Mean	St. Dev	Mean	St. Dev	Mean	St. Dev	
*Outcome variable*							
Mini-tiller adoption (1 = adopters, 0 = non-adopters)	0.375	0.484	0.411	0.492	0.184	0.389	−55.3***
*Household attributes*							
Farm size of the household (ha)	0.471	0.477	0.500	0.500	0.318	0.285	−36.4***
Age of the household head (years)	48.675	10.961	49.346	10.708	45.215	11.618	−8.4***
Education of the household head (years)	5.711	4.375	6.111	4.354	3.650	3.887	−40.3***
Farming experience (years)	25.962	11.836	26.234	11.704	24.558	12.439	−6.4*
Household size (no)	5.693	2.070	5.779	2.122	5.252	1.719	−9.1***
Caste of the household (1 = non-marginalized, 0 = marginalized)	0.532	0.499	0.556	0.497	0.405	0.492	−27.2***
Occupation of the household head (1 = farming, 0 = others)	0.602	0.490	0.577	0.494	0.730	0.445	26.6***
Households off-farm income (’000 NPR)	296.866	260.571	294.087	274.859	311.202	168.534	5.8
Nearest input market distance (km)	8.137	8.288	7.546	7.893	11.182	9.546	48.2***
Members out-migrated per household (no)	0.336	0.563	0.295	0.551	0.546	0.580	85.2***
Membership in groups or cooperatives (1 = yes, 0 = no)	0.704	0.457	0.742	0.438	0.509	0.501	−31.4***
Difficult in finding labors (1 = yes, 0 = no)	0.687	0.464	0.700	0.458	0.620	0.487	−11.5**
Total number of livestock holdings (TLU)	2.091	1.317	2.168	1.362	1.690	0.961	−22.1***
Difficulty in finding draft animals (1 = yes, 0 = no)	0.260	0.439	0.271	0.445	0.202	0.403	−25.3*
Household food security status (1 = food secured, 0 = food in-secured)	0.369	0.483	0.384	0.487	0.288	0.454	−24.9**
Grow rice (1 = yes, 0 = no)	0.622	0.485	0.629	0.483	0.583	0.495	−7.3
Grow maize (1 = yes, 0 = no)	0.739	0.439	0.746	0.436	0.706	0.457	−5.4
Grow wheat (1 = yes, 0 = no)	0.304	0.460	0.320	0.467	0.221	0.416	−31.0***
Grow vegetables (1 = yes, 0 = no)	0.402	0.491	0.409	0.492	0.368	0.484	−10.0

***, **, * significantly different at 1%, 5%, and 10% level across MH-HHs and FH-HHs. Rate of exchange of 1 $ of US = 104 Nepalese Rupees during the time of data collection year [77]. TLU represents standard units to measure the aggregated livestock [78]. St. Dev indicates the standard deviation of the sample mean.

In the survey districts, the mean landholding of the sample farmers is around 0.47 ha, indicating the prevalence of smallholder farming. The MH-HHs have significantly larger farm size (0.50 ha) than FH-HHs (0.32 ha). Earlier studies have also reported a smaller farm size for FH-HHs [47,[49], [50], [51], [52]], which could be a reason for the low mini-tiller adoption among FH-HHs. Although FH-HHs have smaller farm sizes, their reliance on agriculture as a source of livelihood is much higher. Our data show that 73% of the FH-HHs reported farming as their primary occupation, compared to 58% for MH-HHs. The FH-HH heads were found to be younger with less formal education (3.7 years), and smaller family size (3.2). A greater percentage of MH-HHs were from the socially non-marginalized caste groups (56%), indicating the dominance of male members among higher caste households.^4^ It should be noted that similar to many parts of India, Nepal has the pervasive caste systems and household heads across these different castes are mostly responsible for household-level decisions [53,54].

In Nepal, due to the higher trend of male labor migration, many households have women as household heads, as they fulfill the role of household headship in the absence of their male counterparts. However, the male migrants still control households from abroad, a situation termed *de-facto* household headship [30,34,40,[55], [56], [57]]. The result shows that 55% of the FH-HHs have a migrant member, while it is 30% in the case of MH-HHs. Moreover, the average annual off-farm income among MH-HHs and FH-HHs is almost similar, in contrast to many other studies that report lesser household non-farm income among FH-HHs [[58], [59], [60]]. Presumably, many FH-HHs have migrant men who send remittances to their dependents back home [34,39]. The general trends of labor out-migration in Nepal has also affected the size of livestock and human power, mainly decreasing the number of draft animals and the labor force [5]. Our result shows that a higher percentage of MH-HHs reported difficulty in finding agricultural laborers and draft animals, which is perhaps due to the reason that most MH-HHs rely on off-farm incomes compared to FH-HHs. It is also evident that in comparison to FH-HHs, the MH-HHs are located closer to the input markets, and a higher percentage of them are affiliated with social networks such as farmer groups and cooperatives, both of which are likely to affect technology adoption, including mini-tillers.

The livestock ownership may also affect mini-tiller adoption, as bullocks are primarily used for land preparation and/or tillage [61]. In the study area, despite having significantly higher livestock holdings among MH-HHs, a higher percentage of them reported difficulty in finding draft animals during land preparation time. It could be due to the increasing trend of keeping milking cattle over draft animals because of an emerging dairy industry [62]. Since the distance to the local market is shorter for MH-HHs, they have a better market opportunity for milk sales. Likewise, in hilly regions of Nepal, bullocks are only used to plow land, and the opportunity cost of raising them is much higher due to lack of feed, fodder, grazing land, small landholdings, and labor shortages [63].

Cropping systems may also affect mini-tiller adoption in the hills of Nepal. The maize-based farming system is common, as 74% of survey participants were found to grow maize in the preceding year. There is an insignificant difference in the case of maize grown, across the headship of the family by gender. Apart from maize, farmers also grow other crops in different seasons [64,65]. While we did not find a significant difference in relation to growing other crops such as rice and vegetables across the gender cohorts, a greater percentage of MH-HHs grew wheat (32%) compared to FH-HHs (22%). This might be one of the causes for higher food security among MH-HHs, even though food security depends on a multitude of factors. Finally, all these observed differences in the household attributes among MH-HHs and FH-HHs may affect mini-tiller adoption decisions. Therefore, the use of gender as a dummy variable in the regression would fail to account for the interactions amongst gender and other household-level attributes, which justifies the use of the ESTER model.

### 4.2 Mini-tiller adoption for male- and female-headed households: a probit model

We estimated the factors influencing the adoption of mini-tiller separately for the MH-HHs and FH-HHs using a probit model, and results are presented in Table 3. Here, the dependent variable is a dichotomous choice discrete variable and assumes the value of 1 if the household adopted a mini-tiller, otherwise zero.

**Table 3:** Determinants of mini-tiller adoption across MH-HHs and FH-HHs in Nepal hills.

Variables	MH-HHs		FH-HHs	
	Coefficient	SE	Coefficient	SE
Model intercept	−2.367***	0.461	−3.028**	1.367
Farm size (ha)	0.018	0.120	−0.776	0.799
Households heads age (years)	0.005	0.010	−0.006	0.040
Years of formal education (years)	0.054***	0.017	0.114*	0.061
Farming experience (years)	0.003	0.008	0.051	0.040
Household size (no)	0.045	0.033	0.114	0.140
Households caste (1 = non-marginalized)	0.430***	0.122	1.079***	0.436
Household heads occupation (1 = farming)	0.209*	0.125	0.654	0.518
Off-farm income (NPR)	3E-07	2E-07	-3E-06*	2E-06
Input market distance (km)	−0.129***	0.014	−0.148***	0.058
Members out-migrated (no)	−0.224*	0.123	0.386	0.374
Membership in groups or cooperatives (1 = yes)	0.397***	0.146	0.108	0.414
Difficult in finding labors (1 = yes)	0.222*	0.131	0.146	0.477
Total number of livestock holdings (TLU)	0.060	0.044	−0.257	0.238
Difficulty in finding draft animals (1 = yes)	0.843***	0.128	0.991***	0.413
Grow rice (1 = yes)	0.915***	0.122	1.254***	0.505
Grow maize (1 = yes)	0.098	0.132	−1.180**	0.554
Grow wheat (1 = yes)	−0.305***	0.124	0.960*	0.572
Grow vegetables (1 = yes)	0.349***	0.120	0.593	0.424
Log likelihood	−341.36		−36.52	
LR-χ^2^	456.60		82.62	
Pseudo R^2^	0.401		0.531	
Model correctly classified adopters and non-adopters (%)	80.14		90.18	
Number of observations	841		163	

***, **, and * indicate significant at 1%, 5%, and 10% levels, respectively. SE: Standard errors.

The results show that years of formal education of the household head, caste, crops grown such as rice, and difficulty in finding draft animals during land preparation, positively influence mini-tiller adoption for both MH-HHs and FH-HHs. However, the input market distance is negatively associated with the probability of mini-tiller adoption for both gender groups, indicating a higher probability of mini-tiller adoption when the input markets are at a closer distance. These findings are consistent with earlier studies that have demonstrated; (i) a higher probability of technology adoption when the input markets are closer [5,66,67], (ii) affiliation with non-marginalized caste groups [68], and (iii) the level of education [69,70]. The coefficient of rice farming (rice crop cultivated) was positive and statistically significant at 1% level indicating that the probability of adoption of mini-tiller is high among farm households who grow rice, probably as it is a labor-requiring crop for land preparation, puddling, seedling up-rooting, and transplantation [5,71,72].

There are some MH-HHs and FH-HH level attributes that affected the mini-tiller adoption decision differently. The MH-HHs with their primary occupation as agriculture have a higher probability of mini-tiller adoption, while it is not the case for the FH-HHs. In MH-HHs, we found that the larger the number of migrated members, the lower is the probability of mini-tiller adoption. Moreover, MH-HHs cultivating vegetables were more likely to adopt mini-tiller, while there was no significant effect of growing vegetables in FH-HHs. In Nepal, in general, vegetable cultivation among smallholders as a cash crop is growing due to the high demand for vegetables in the urban areas and market centers. Since MH-HHs are located closer to markets, they could have adopted mini-tillers to reduce the cost of cultivation [73,74]. Moreover, MH-HHs’ affiliation with groups or cooperatives increases the mini-tiller adoption, while there were no significant effects of institutional networks among FH-HHs.

Nevertheless, households’ non-farm income has a negative relationship with mini-tiller adoption for the FH-HHs, while the income effect is not significant for MH-HHs. It ought to be noted that the number of migrant members in FH-HHs is higher; hence, off-farm income is also significantly higher potentially due to remittance money they have received from their male counterparts. The higher off-farm income has created a situation where farmers do not want to cultivate agricultural land anymore, leaving the cultivable land fallow [41]. This could be the reason for the negative effect of off-farm income for mini-tiller adoption. Likewise, the probability of mini-tiller adoption is positively associated with wheat-growing FH-HHs. Wheat is considered an important crop for food security, and FH-HHs are relatively food insecure compared to MH-HHs. This could have motivated them to adopt a mini-tiller. Surprisingly, mini-tiller adoption is negatively associated with maize growing FH-HHs, although FH-HHs are more food insecure. This may be because the use of maize as food is decreasing due to changing consumption patterns and increasing off-farm incomes, the high cost of cultivation, and the lack of markets and potential remuneration from technology adoption [62,75].

### 4.3 Impact of the gender of the household head on mini-tiller adoption

Results of the impact of the household head's gender for the MH-HHs and FH-HHs, along with the base heterogeneity estimate effects from the ESTER model as specified in Equations (6), (7), (8), (9), are presented in Table 4. The result displays that if the MH-HHs were assigned to the same level of coefficients that FH-HHs are entitled to, their mini-tiller adoption rate would have significantly (*P < 0.001*) decreased by 13%. However, if FH-HHs were assigned the same level of the coefficient that MH-HHs are entitled to, their mini-tiller adoption rate would have increased by 5% (*P < 0.10*). This indicates an improvement in the mini-tiller adoption rate for the FH-HHs when they have similar household attributes coefficient as of MH-HHs. This finding is consistent with previous gender-related literature [[45], [46], [47],^76^], highlighting the need to foster gender equality by minimizing the differences between MH-HHs and FH-HHs to enhance the adoption of small-scale mechanization in Nepal hills.

**Table 4:** Average probability of mini-tiller adoption, treatment, and heterogeneity effects for MH-HHs and FH-HHs.

Household type	MH-HHs	FH-HHs	Treatment effects
Male-headed households	0.409	0.283	0.127*** (0.000)
Female-headed households	0.238	0.185	0.053* (0.095)
Heterogeneity effects	0.171*** (0.000)	0.097*** (0.000)	

***, **, and * indicate significant at 1%, 5%, and 10% levels, respectively. Numbers in parentheses indicate *p*-values, while the number without parentheses are the mini-tiller adoption rates.

MH-HHs could, nonetheless, be different due to the inherent skills (for example the risk-bearing ability, physical capacity, managerial skills, etc.) and different in accessibility to social networks of importance for technology diffusion that may affect the mini-tiller adoption, even if FH-HHs would have the same returns in coefficients, which can be detected from the base heterogeneity effects. The analysis using Eqs. (8), (9) shows that the base heterogeneity effects of mini-tiller adoption for MH-HHs is 17% *(P < 0.001*; Table 4). It indicates that the average mini-tiller adoption by FH-HHs would have been considerably lower than the MH-HHs, even if the returns in coefficients would have been the same for MH-HHs.

Moreover, the currently observed difference in the mini-tiller adoption rate between MH-HHs and FH-HHs is around 55% (Table 2). However, the base heterogeneity effects of the mini-tiller adoption rate for FH-HHs is 9% (*P < 0.001*; Table 4). This result suggests that the average probability of mini-tiller adoption by MH-HHs would have been only 9% higher than FH-HHs, if the MH-HHs’ coefficients would have been assigned for FH-HHs, which is due to the existence of the unobserved heterogeneity effects.

Furthermore, we stratified our dataset into food secure and insecure household groups to get insights on differential mini-tiller adoption rates for the MH-HHs and FH-HHs, and the results are shown in Table 5. For the MH-HHs, in both food secure and insecure groups, if MH-HHs are assigned to the same level of coefficients that FH-HHs are entitled to, their mini-tiller adoption rates would have decreased significantly, respectively by 16% (*P < 0.001*) and 10% (*P < 0.001*). This reflects the low propensity of mini-tiller adoption for food-insecure households, even after switching the coefficients. This is potentially due to limited access to resources and high poverty among food insecure MH-HHs.

**Table 5:** Heterogeneous probability of mini-tiller adoption across gender-differentiated food secure and insecure households.

Household type	Food secure households			Food insecure households		
	MH-HHs	FH-HHs	Treatment effects	MH-HHs	FH-HHs	Treatment effects
MH-HHs	0.507	0.342	0.165*** (0.000)	0.348	0.246	0.103*** (0.000)
FH-HHs	0.346	0.313	0.032 (0.645)	0.194	0.134	0.060** (0.051)
Heterogeneity effects	0.162*** (0.001)	0.029 (0.584)		0.154*** (0.000)	0.112*** (0.000)	

***, **, and * indicate significant at 1%, 5%, and 10% levels, respectively. Numbers in parentheses indicate *p*-values, while the number without parentheses are the mini-tiller adoption rates.

Nevertheless, if food secure FH-HHs were assigned with the same level of coefficients of MH-HHs, the returns in the coefficient would not bring any significant changes in the mini-tiller adoption rates. For the FH-HHs among the food insecure group, however, the return in coefficients would have increased the mini-tiller adoption rate significantly by 6% (*P < 0.05*). Hence, our findings highlight the need to prioritize food insecure FH-HHs by closing the gender gap between MH-HHs and FH-HHs to enhance the adoption of small-scale mechanization in Nepal and similar hill landscapes in South Asia with labor shortages and high rate of out-migration.

## 5 Conclusion and policy implications

Currently, the Nepalese agricultural sector is suffering from agricultural labor scarcity due to recent trends of male out-migration that has increased the wage rates in rural areas. Women increasingly have multiple roles and responsibilities, including additional agricultural work in the absence of their male counterparts. In subsistence agricultural systems where most farm operations are manual, increasing labor costs, as well as a reduction in animal traction, are leading to more land being left fallow and to lower productivity. In this situation, farm mechanization can be a viable option to counterbalance the effects of labor scarcity and enhance agricultural productivity.

Our analysis, using ESTER framework and primary survey data from the hilly region in Nepal, shows that there are substantial differences in the adoption of small-scale farm mechanization between MH-HHs and FH-HHs, and these differences are larger when we compared with food-secure and food-insecure FH-HHs. We find that differences in adopting mini-tillers between MH-HHs and FH-HHs is large and is mostly elucidated by the unobserved sources of heterogeneity between them. As a result, the prospect of adopting mini-tillers for female-headed households would remain lower even after having the same level of attributes for the MH-HHs and FH-HHs. Hence, it is recommended that to maintain gender equality in agricultural mechanization and to enhance agricultural productivity, policymakers should aim at providing equal opportunities through female-targeted farm mechanization policies and programs. This will enhance the level of small-scale mechanization in the hilly region of Nepal and similar landscape in South Asia.

## CRediT authorship contribution statement

**Gokul P. Paudel:** Conceptualization, Methodology, Data curation, Formal analysis, Writing - original draft, Writing - review & editing. **Hom Gartaula:** Conceptualization, Methodology, Writing - review & editing. **Dil Bahadur Rahut:** Conceptualization, Methodology, Writing - review & editing. **Peter Craufurd:** Conceptualization, Methodology, Writing - review & editing.

## Declaration of competing interest

We declare to have no conflict of interest.

## Footnotes

1 Mini-tillers are small tractors (5–9 horsepower) that are adopted primarily for agricultural land-preparations and soil tillage. These small machines are used for maize, rice, wheat, and vegetables land preparation. It is considered as the scale-appropriate farm machinery as it fits to the socio-economic and land typologies in the hilly areas of Nepal and is also women friendly technology. For details on the mini-tillers see Refs. [79,80].

2 The selected six districts were Doti (Far-western region), Surkhet (Mid-western region), Palpa (Western region), Nuwakot and Kavre (Central region), and Illam (Eastern region).

3 However, the districts were selected purposively based on high level of mini-tiller adoption. Therefore, this adoption rate represents only for the sample data and not for the overall hilly region.

4 Nepal has a pervasive caste system where Brahmin and Chettri are considered non-marginalized, while Vaishya and Shudra are considered marginalized castes.
